# Post-COVID-19 syndrome and health-related quality of life after hospital discharge

**DOI:** 10.11606/s1518-8787.2025059006650

**Published:** 2025-12-01

**Authors:** Roseany Patricia Silva Rocha, Amanda Cristina de Souza Andrade, Ana Paula Muraro

**Affiliations:** I Universidade Federal de Mato Grosso. Instituto de Saúde Coletiva. Cuiabá, MT, Brazil

**Keywords:** Post-COVID-19 Syndrome, Hospital, Quality of Life

## Abstract

**OBJECTIVE:**

To analyze the association between the presence of post-COVID-19 syndrome after six months and health-related quality of life 6 and 12 months after hospital discharge.

**METHOD:**

An ambidirectional cohort study was conducted with individuals discharged from three of the main hospitals in Cuiabá (in the state of Mato Grosso) between October 2021 and March 2022. After collecting data from medical records, individuals were interviewed via telephone 6 months (n = 189) and 12 months (n = 159) after hospital discharge, evaluating the presence of at least one persistent post-COVID-19 symptom at 6 months (post-COVID-19 syndrome) and health-related quality of life (EQ-5D-3L) at 6 and 12 months, as well as specific dimensions (mobility, self-care, usual activities, pain/discomfort and anxiety/depression). The association was assessed via Poisson regression with robust variance, adjusted for sociodemographic, health, and hospitalization characteristics.

**RESULTS:**

Of the individuals assessed, 88.4% answered that they had COVID-19 symptoms in the interview after 6 months. At 6 and 12 months after hospital discharge, 55.0% and 74.2% of individuals, respectively, had some impairment in quality of life. In the multiple model, post-COVID-19 syndrome remained associated with having any quality of life issue (RR = 2.43; 95%CI: 1.06–5.57) and specifically with the anxiety/depression domain (RR = 2.74; 95%CI: 1.08–7.01) at 6 months after discharge. The association was no longer significant after 12 months.

**CONCLUSION:**

These results show the long-term negative repercussions of post-COVID-19 syndrome on cognitive, emotional, and physical functions, exposing the negative impact on the quality of life of those affected.

## INTRODUCTION

The COVID-19 pandemic, declared by the World Health Organization (WHO) in March 2020, represented a serious threat to global public health, with more than 712,000 deaths in Brazil from the disease recorded in Brazil^1^. With the advance of new studies, it has become clear that SARS-CoV-2 does not only cause respiratory problems, but can trigger a syndrome that affects multiple organs. In addition to the initial acute infection, several lasting and new symptoms of COVID-19 have been identified^4^.

More than a year after the onset of this crisis, in October 2021, WHO officially announced the existence of a “post-COVID-19 syndrome” as a specific clinical condition^5^, describing a diverse set of symptoms that persist for more than 12 weeks after infection, with no other diagnostic explanation, the most frequent symptoms being fatigue, dyspnea, joint pain, memory loss, hair loss, and anxiety^6^. The WHO has presented an official clinical definition, using the term “post-COVID-19 condition”^4,5^, and the US Centers for Disease Control and Prevention uses the term “post-COVID conditions (PCC)”^10^. Other nomenclatures are also found in the scientific literature, such as “long COVID,” “post-acute COVID-19,” “long-term effects of COVID,” “post-acute COVID syndrome,” “chronic COVID syndrome,” among others^3,11^. In Brazil, the Ministry of Health (MoH) opted for the term “post-Covid conditions” to standardize technical documents and guide health professionals on the subject^12^.

The proportion of individuals with post-COVID-19 syndrome after recovery from the acute phase of COVID-19 has varied across studies^7,9,11^. A systematic review and meta-analysis with 41 meta-analyzed studies from 16 countries, mainly China and the United States, with adult patients with more than six months of follow-up identified an overall prevalence of 43.0% of post-covid conditions, being higher among patients who required hospitalization (54.0%) compared to those who were not hospitalized (34.0%). Fatigue was the most commonly reported symptom, followed by memory problems. Only one Brazilian study was included in the review, with hospitalized patients and an average of 6 months of follow-up, specifically assessing cognitive and psychiatric symptoms^11^.

Health-related quality of life (HRQoL) is defined as an individual’s subjective assessment of the impact of their health on different aspects of their life. It is a measure of physical, psychological and social well-being, influenced by individual experiences, beliefs, expectations and perceptions^13^.

In the dynamic context of the pandemic, HRQoL has aroused great interest in the scientific community, especially regarding the impact of the disease on patients’ physical and cognitive function and overall satisfaction with life^14^. Recent studies have assessed quality of life among adults with persistent symptoms, indicating a significant reduction in quality of life, particularly with regard to increased symptoms of anxiety, depression, and cognitive difficulties^8,17^.

A systematic review and meta-analysis of studies with follow-up times ranging from one to six months found that 59% of patients with persistent symptoms reported poor quality of life^17^. The most prevalent persistent symptoms were fatigue, dyspnea, anosmia, arthralgia, headache, and sleep disturbances, as well as impacts on mental health. A meta-regression analysis showed that poor quality of life was significantly higher among patients who required ICU admission during acute infection^17^.

In the national context, results found by Ida et al.^8^in adult patients aged 40 to 59 years indicate that those with persistent fatigue, arthralgia and dyspnea for more than 12 months had a worse quality of life compared to those who did not have persistent COVID-19 symptoms.

Another crucial point is that, despite the increase in evidence on the possible impacts of COVID-19 on quality of life, the relationship between post-COVID-19 syndrome and HRQoL is still not fully understood. Many existing studies have not considered adjustments for potential confounders, such as sociodemographic characteristics, pre-existing clinical conditions, and variables related to hospitalization^8,15,19^. The lack of adjustments can compromise the validity of the findings and lead to biased interpretations of the association between the syndrome and HRQoL. In this sense, this study seeks to fill this gap, offering a more robust and accurate analysis of the subject.

Therefore, this study proposes to analyze the association between the presence of post-COVID-19 syndrome after 6 months and health-related quality of life 6 and 12 months after hospital discharge, adjusting for sociodemographic variables and characteristics related to hospitalization.

## METHODS

### Study Population

This is an ambidirectional cohort study, conducted with patients confirmed with COVID-19, living in Cuiabá and Várzea Grande (which make up the state’s metropolitan region), who were admitted to public and private hospitals in Cuiabá and whose case was closed as a hospital discharge from October 2021 to March 2022.

The cases were identified by IndicaSUS, a system implemented by the state of Mato Grosso^20^, via access to the Cuiabá Municipal Health Department. After collecting data from medical records, the individuals were interviewed by telephone 6 and 12 months after discharge from hospital.

Of the seven hospitals in Cuiabá, only three allowed access to patient records. Eligible individuals were those aged 18 or over, confirmed cases (by polymerase chain reaction [PCR], rapid test or antigen test, or confirmation by imaging tests) and those who had been discharged from hospital according to information recorded in IndicaSUS. Exclusion criteria were: patients living in nursing homes and patients with communication difficulties (aphasia, cognitive impairment, and severe hearing loss).

We identified 998 confirmed cases of COVID-19 among residents of Cuiabá and Várzea Grande who had their case closed in the capital’s hospitals during the period in question. Of these, 689 were discharged from hospital (69.0%), 277 in the three hospitals that allowed access to medical records (40.2% of all adult patients discharged in the period). During the collection of medical records, ten patients were excluded because they lived in a nursing home and eight who, despite being recorded in IndicaSUS as discharged from hospital, died during hospitalization. Therefore, 259 individuals remained eligible for the study, whose data was collected from the medical records, the retrospective phase of the cohort. The details of the follow-up of the study population can be found in Rocha et al.^9^For this analysis, three individuals were excluded due to lack of information in the medical records, and 256 individuals were analyzed.

### Data Collection

The prospective stages consisted of two evaluations using telephone interviews. At 6 months, 190 individuals were interviewed (73.3% of the baseline) and 160 individuals were interviewed at 12 months. A total of 28 deaths were identified through the Mortality Information System, consulted by the Cuiabá Municipal Health Department. In order to analyze the data, it was necessary to exclude one patient who did not provide complete answers to the health-related quality of life questionnaire in the interview six months after hospital discharge, so 189 patients were considered at six months and 159 at 12 months.

The interviews were individual, conducted by telephone using a structured questionnaire with questions on demographic and socioeconomic characteristics, housing and living conditions, health characteristics and hospitalization data, as well as on persistent symptoms from the acute phase of COVID-19 or new ones up to the time of the interview.

### Exposure and Outcome Variables

The outcome variable of this study was HRQoL, assessed by the three-level version of the EQ-5D questionnaire (EQ-5D-3L) validated for assessing health-related quality of life in the Brazilian population^21^, which comprises five domains: mobility, self-care, usual activities, pain/discomfort, and anxiety/depression. The participant describes the severity levels of each dimension on a scale of 1 to 3, in which 1 indicates no problem, 2 indicates some problem, and 3 indicates extreme problem. Therefore, 243 different combinations of health states are possible.

Each combination of answers in the five dimensions generates a five-digit health status profile, such as 11111 (no problems in any dimension) or 22332 (some problems with mobility, self-care, usual activities, etc.). The combination can be translated into a single summary index value that ranges from -0.1755 (worse than dead) to 1.000 (perfect HRQoL) and is weighted according to Brazilian values and preferences regarding health outcomes^21^. The EQ-5D-3L score was assessed continuously and categorically, with the latter being dichotomized as 1.0 for no problem and less than 1.0 for any HRQoL problem^22^. The domains were organized into: mobility, self-care, common activities, pain/illness, anxiety, and depression. The answers for each domain were defined as “no problem” or “some problem,” according to the score previously described.

The exposure variable of interest was post-COVID-19 syndrome, considered to be the presence of persistent symptoms at the time of the interview at 6 and 12 months after hospital discharge^7^. They were asked about 24 classes of symptoms present in the acute phase of COVID-19 and after 6 and 12 months from hospital discharge, this list of symptoms was constructed from the literature^23^. The symptoms were classified as muscular, neuropsychiatric, dermatological, cardiovascular, and pulmonary and are detailed in the article by Rocha et al.^9^

### Adjustment Variables

The sociodemographic and economic variables analyzed were: gender (female; male), age group (18 to 29 years; 30 to 49 years; 50 to 59 years; 60 years or more), race/color (white; Mixed-race/Brown; Black; Yellow/Indigenous; and unknown), education (completed primary school; completed secondary school; completed higher education or more), monthly income (considering the minimum wage in force when the data was collected R$ 1.212.00) per capita (up to 2 wages; 2 to 3 minimum wages; 3 or more minimum wages), current employment status at six months (yes; no), housing (average; good/very good). With regard to health and hospitalization characteristics, the self-reported number of vaccine doses received before hospitalization (1; 2; 3 or 4 doses) was considered, and the variables obtained from hospital records were: length of hospital stay (days of hospitalization assessed on average and classified into tertiles; first tertile from 1 to 6 days, second tertile from 7 to 12 days, third tertile from 13 to 134 days), intensive care unit (ICU) stay (yes; no), need for mechanical ventilation (yes; no).

To assess the number of comorbidities, self-reported diagnoses of hypertension, diabetes mellitus and heart disease, asthma/bronchitis, cancer, kidney disease, lung disease, obesity, mental disorder or depression or other chronic diseases were considered. To analyze the comorbidities individually, hypertension, diabetes and heart disease were considered, as they were the most frequent among the comorbidities. To classify obesity, self-reported weight and height were taken into account in the interviews carried out 6 months after hospital discharge to calculate the body mass index; only three individuals were unable to provide information and remained as missing data. Obesity was classified as 30kg/m^2^ or more^24^. In addition, the co-occurrence of hypertension, diabetes mellitus, heart disease, and obesity was also considered, classified as: no comorbidities, one or two comorbidities, and three or more comorbidities.

The variable of co-occurrence of risk factors for chronic non-communicable diseases (CNCD), assessed 12 months after hospital discharge, was made up of the combination of: smoking, excessive alcohol consumption, insufficient physical activity, and regular consumption of unhealthy foods. Exposure to smoking was defined as individuals who reported being current or former smokers, and excessive alcohol consumption was considered to be those who reported consuming more than five drinks for men and four for women on a single occasion^25^. Physical activity was assessed using the International Physical Activity Questionnaire (IPAQ) short version, with less than 150 minutes per week considered insufficient^26^. The presence of at least one risk factor for CNCD was considered as inadequate.

### Data Analysis

The variables included in the adjusted model were identified using a directed acyclic graph (DAG) with the aid of the Daggity program, version 3.016, identifying the minimum set of adjustment variables to be used in multiple models to estimate the association between these two variables. The minimum adjustment variables identified included: gender, age group, education level, race/color, length of hospital stay, ICU stay, number of comorbidities and co-occurrence of risk factors for NCDs (Figure).

**Figure:**
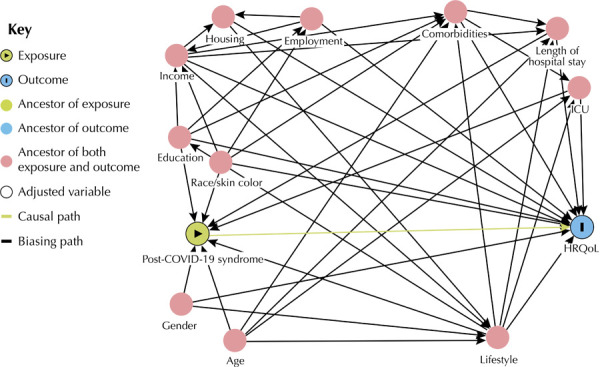
Directed acyclic graph representing hypotheses about the relationships between exposure, outcome and other covariables and the post-COVID-19 condition. Cuiabá (MT). 2024.

ICU: intensive care unit; HRQoL: health-related quality of life; CNCD: chronic non-communicable diseases.

Note: comorbidities (no comorbidities. 1 or 2 comorbidities and 3 or more comorbidities). The term co-occurrence of risk factors for NCDs was used to replace lifestyle.

The analyses were carried out using Stata software, version 16.0. The follow-up rate was estimated and the distribution of sociodemographic variables between participants and non-participants was compared in order to check for possible selective losses. A descriptive analysis was carried out, calculating absolute and relative frequencies for categorical variables and the mean and standard deviation (SD) for quantitative variables. To compare the continuous HRQoL according to the explanatory variables, the t-test for independent samples (comparison of two groups) or ANOVA (comparison of three groups or more) were used, if the normality assumption was met. Otherwise, the Mann-Whitney and Kruskall Wallis non-parametric tests were used, respectively. Pearson’s chi-square test was used to assess the association between dichotomized HRQoL and the explanatory variables. For the multiple analysis, the risk ratios (RR) and respective p-values and 95% confidence intervals (95%CI) were estimated using Poisson regression with robust variance to assess the association between the independent variable of interest (post-COVID-19 syndrome) and the outcome (quality of life and its domains).

### Ethical Considerations

This study was approved by the Ethics Committee for Research with Human Beings in the Health Area of the Universidade Federal do Mato Grosso-Campus Cuiabá (Opinion no. 5.415.255/2022, dated May 18, 2022). All participants signed an informed consent form.

## RESULTS

A total of 189 patients (73.0% of those eligible) were assessed 6 months after hospital discharge (mean: 6.29 months, SD = 1.24) and 159 patients (61.4% of those eligible) 12 months after hospital discharge (mean: 13.17 months, SD = 1.11). At 6 months from hospital discharge, 55.0% of individuals were classified as having any HRQoL problem. This proportion was 74.2% 12 months after discharge (data not shown in table).

The follow-up rate remained lower among men (64.1% and 53.3%) compared to women (82.3% and 69.8%) aged 60 and over (64.4% and 43.2%) when compared to younger age groups and among those of Black race/skin color (53.3% and 46.6%) after 6 and 12 months from hospital discharge, respectively. There was no significant difference in the rate according to race/color, length of stay and whether admitted to the ICU (Table 1).

**Table 1:** Sociodemographic and hospitalization characteristics and 6- and 12-month follow-up rates. Cuiabá (MT), 2024.

	Baseline (n = 256)	6 months (n = 89)		12 months (n = 159)	
	n (%)	n (%)	Follow-up rate (%)	n (%)	Follow-up rate (%)
Sex					
Male	120 (46.8)	77 (40.7)	64.1	64 (40.2)	53.3
Female	136 (53.2)	112 (59.3)	82.3	95 (59.7)	69.8
p-value		< 0.01		0.02	
Age group					
18 to 29 years old	29 (11.3)	23 (12.1)	79.3	20 (12.5)	68.9
30 to 49 years old	74 (28.9)	61 (32.2)	82.4	59 (37.1)	79.7
50 to 59 years old	35 (13.6)	29 (15.3)	82.8	29 (18.2)	82.8
≥ 60 years old	118 (46.1)	76 (40.2)	64.4	51 (32.1)	43.2
p-value		0.01		< 0.01	
Race/skin color					
White	42 (16.4)	36 (19.0)	85.7	34 (21.4)	80.9
Brown/Mixed-race	166 (64.8)	120 (63.5)	72.3	96 (60.4)	57.8
Black	15 (5.8)	8 (4.2)	53.3	7 (4.4)	46.6
Yellow	15 (5.8)	11 (5.8)	73.3	10 (6.3)	66.6
Ignored	18 (7.0)	14 (7.4)	77.7	12 (7.5)	66.6
p-value		0.15		0.30	
Length of stay (days) tertile					
1^st^ tertile (1 to 6 days)	91 (35.5)	73 (38.8)	80.2	64 (40.2)	70.3
2^nd^ tertile (7 to 12 days)	88 (34.5)	61 (32.4)	69.3	49 (30.0)	55.7
3^rd^ tertile (13 to 134 days)	77 (30.2)	54 (28.7)	70.1	46 (29.1)	59.7
p-value		0.14		0.61	
Admitted to ICU					
Yes	77 (30.1)	57 (30.1)	74.0	45 (28.3)	58.4
No	179 (69.9)	132 (69.8)	73.7	114 (71.7)	63.6
p-value		0.96		0.20	

ICU: intensive care unit.

Six months after hospital discharge, 88.4% of individuals were classified as having post-COVID-19 syndrome. The proportion of individuals who reported any quality of life problems at 6 and 12 months after hospital discharge was higher (59.3% and 77.0%) among those with post-COVID-19 syndrome at 6 months, compared to those without this condition (22.7% and 55.0%, respectively), with a significant difference (p-value < 0.01 and p-value = 0.03, respectively). The mean and proportion of any HRQoL problem was significantly higher at 6 months among individuals with lower monthly income and who required mechanical ventilation during hospitalization; and at 12 months among those with lower education, the other variables showed no significant difference (Table 2).

**Table 2:** Mean score and proportion of any HRQoL problem, according to sociodemographic and hospitalization-related factors 6 and 12 months after discharge. Cuiabá (MT), 2024.

Characteristic	6 months (n = 189)			12 months (n = 159)		
	General	HRQoL^a^		General	HRQoL^a^	
	n (%)	Any problem^a^ n (%)	Mean (SD)	n (%)	Any problem n (%)	Mean (SD)
Post-COVID-19 syndrome at 6 months^b^						
Yes	167 (88.4)	99 (59.3)	0.84 (0.16)	139 (87.4)	107 (77.0)	0.84 (0.19)
No	22 (11.6)	5 (22.7)	0.91 (0.22)	20 (12.6)	11 (55.0)	0.77 (0.19)
p-value		< 0.01	0.08		0.03	< 0.01
Sex						
Male	77 (41.0)	38 (49.3)	0.86 (0.19)	64 (40.2)	44 (68.7)	0.79 (0.20)
Female	112 (59.0)	66 (58.9)	0.84 (0.16)	95 (59.7)	74 (77.9)	0.77 (0.18)
p-value		0.44	0.39		0.12	0.41
Age group at hospitalization						
18 to 29 years	23 (12.1)	13 (56.5)	0.89 (0.23)	20 (12.6)	16 (80.0)	0.80 (0.14)
30 to 49 years	61 (32.3)	31 (50.8)	0.86 (0.23)	59 (37.1)	38 (64.4)	0.80 (0.18)
50 to 59 years	29 (15.3)	18 (62.1)	0.84 (0.26)	29 (18.2)	20 (68.9)	0.78 (0.21)
≥ 60 years	76 (40.0)	42 (55.2)	0.84 (0.21)	51 (32.1)	44 (86.3)	0.74 (0.20)
p-value		0.62	0.64		0.29	0.34
Race/skin color						
White	36 (18.9)	20 (55.5)	0.84 (0.18)	34 (21.4)	25 (73.5)	0.76 (0.22)
Brown/Mixed-race	120 (63.2)	66 (55.0)	0.85 (0.17)	96 (60.4)	69 (71.9)	0.78 (0.19)
Black	8 (4.2)	4 (50.0)	0.86 (0.15)	7 (4.4)	5 (71.4)	0.86 (0.09)
Yellow	11 (5.8)	6 (54.5)	0.83 (0.16)	10 (6.3)	10 (100)	0.72 (0.11)
Ignored	14 (7.4)	8 (57.1)	0.86 (0.13)	12 (7.5)	9 (75.0)	0.76 (0.19)
p-value		0.57	0.97		0.69	0.64
Schooling						
Up to complete primary school	106 (55.8)	58 (54.7)	0.84 (0.17)	80 (50.3)	69 (86.2)	0.73 (0.20)
Complete secondary school	51 (26.8)	25 (49.0)	0.87 (0.15)	49 (30.8)	30 (61.2)	0.82 (0.18)
Complete university degree or more	32 (16.9)	21 (65.6)	0.83 (0.19)	30 (18.8)	19 (63.3)	0.83 (0.14)
p-value		0.39	0.40		< 0.01	< 0.01
Monthly income at 6 months^c^						
Up to 2 MW	59 (32.1)	39 (66.1)	0.81 (0.17)	48 (31.2)	43 (89.6)	0.75 (0.14)
2 to 3 MW	58 (30.6)	26 (44.8)	0.89 (0.14)	45 (28.3)	29 (64.4)	0.79 (0.21)
≥ 3 MW	66 (35.8)	34 (51.5)	0.87 (0.13)	60 (37.7)	41 (68.3)	0.19 (0.21)
p-value		0.03	0.03		0.12	0.49
Housing						
Average	10 (5.3)	6 (60.0)	0.80 (0.22)	10 (100.0)	8 (80.0)	0.07 (0.18)
Good/very good	179 (94.7)	98 (54.7)	0.85 (0.17)	149 (83.2)	110 (73.8)	0.78 (0.19)
p-value		0.49	0.39		0.66	0.63
Current employment status at 6 months						
Yes	78 (41.2)	34 (43.6)	0.89 (0.13)	73 (93.6)	46 (63.0)	0.81 (0.18)
No	111 (58.7)	70 (63.1)	0.82 (0.18)	86 (77.5)	72 (83.7)	0.75 (0.19)
p-value		< 0.01	0.04		< 0.01	0.02
Length of stay (days) – tertile						
1^st^ tertile (1 to 6 days)	74 (39.1)	41 (55.4)	0.86 (0.16)	64 (40.2)	44 (69.8)	0.78 (0.20)
2^nd^ tertile (7 to 12 days)	61 (32.4)	31 (50.8)	0.85 (0.17)	49 (30.8)	39 (79.6)	0.75 (0.20)
3^rd^ tertile (13 to 134 days)	54 (28.7)	32 (59.2)	0.83 (0.18)	46 (28.9)	35 (76.1)	0.79 (0.16)
p-value		0.12	0.63		0.14	0.51
Required ICU admission						
Yes	57 (30.0)	32 (56.1)	0.83 (0.20)	45 (28.3)	34 (75.5)	0.79 (0.18)
No	132 (69.8)	72 (54.5)	0.86 (0.16)	114 (71.7)	84 (73.7)	0.77 (0.19)
p-value		0.54	0.35		0.29	0.56
Required mechanical ventilation						
Yes	9 (4.8)	7 (77.8)	0.07 (0.15)	8 (5.0)	7 (87.5)	0.76 (0.21)
No	180 (95.2)	97 (53.9)	0.89 (0.12)	151 (95.0)	111 (73.5)	0.79 (0.19)
p-value		< 0.01	< 0.01		0.83	0.69

HRQoL: health-related quality of life; SD: standard deviation; MW: minimum wage; ICU: intensive care unit.

^a^ HRQoL - no problems = 1.000; any problem < 1.000.

^b^ Post-COVID-19 syndrome classified as the presence of any persistent symptom 6 months after hospital discharge.

^c^ The current wage of R$1,212.00 was considered. Minimum wage only at 6 months from hospital discharge.

At six months from hospital discharge, there was a lower mean HRQoL and a higher proportion of any HRQoL problem among individuals with hypertension, heart disease and two or more comorbidities when hospitalized. However, these differences did not remain significant when assessed 12 months after hospital discharge (Table 3).

**Table 3:** Mean score and proportion of any HRQoL problem according to health conditions 6 and 12 months after hospital discharge. Cuiabá (MT), 2024.

Variables	6 months (n = 189)			12 months (n = 159)		
	General	HRQoL^a^		General	HRQoL^a^	
	n (%)	Any problem^a^ n (%)	Mean (SD)	n (%)	Any problem n (%)	Mean (SD)
Comorbidities						
Hypertension						
No	78 (49.0)	39 (50.0)	0.88 (0.15)	72 (45.2)	49 (68.1)	0.79 (0.18)
Yes	111 (69.8)	65 (58.5)	0.83 (0.18)	87 (54.7)	69 (79.3)	0.76 (0.19)
p-value		0.24	0.02		0.10	0.23
Diabetes mellitus						
No	125 (66.1)	66 (52.8)	0.87 (0.15)	111 (69.8)	80 (72.1)	0.78 (0.19)
Yes	64 (33.8)	38 (59.4)	0.82 (0.19)	48 (30.2)	38 (79.2)	0.77 (0.17)
p-value		0.39	0.06		0.67	0.87
Obesity^b^						
No	179 (94.7)	97 (54.2)	0.86 (0.18)	151 (94.9)	113 (74.8)	0.79 (0.17)
Yes	10 (20.7)	7 (70.0)	0.83 (0.16)	8 (5.0)	5 (62.5)	0.76 (0.23)
p-value		0.32	0.32		0.43	0.35
Heart disease						
No	181 (95.7)	96 (53.0)	0.86 (0.16)	151 (94.9)	110 (72.8)	0.78 (0.19)
Yes	8 (4.2)	8 (100.0)	0.63 (0.22)	8 (5.0)	8 (100.0)	0.73 (0.12)
p-value		< 0.01	< 0.01		0.30	0.47
Number of comorbidities on admission						
No comorbidities	53 (27.9)	24 (45.3)	0.89 (0.16)	50 (31.4)	34 (68.0)	0.79 (0.20)
1 or 2	124 (65.6)	70 (56.4)	0.84 (0.16)	98 (61.6)	73 (74.5)	0.78 (0.17)
3 or more	12 (6.3)	10 (83.3)	0.72 (0.22)	11 (6.9)	11 (100.0)	0.64 (0.26)
p-value		< 0.01	< 0.01		0.08	0.04
Co-occurrence of risk factors for CNCD^c^						
No	-	-	-	22 (13.8)	14 (63.6)	0.79 (0.22)
Yes	-	-	-	137 (86.1)	104 (75.9)	0.77 (0.18)
p-value					0.22	< 0.32
Vaccination						
1 dose	32 (16.9)	19 (59.4)	0.87 (0.11)	30 (18.8)	21 (70.0)	0.79 (0.20)
2 doses	78 (41.2)	40 (51.3)	0.86 (0.16)	67 (42.1)	47 (70.1)	0.78 (0.20)
3 or 4 doses	62 (32.8)	35 (56.4)	0.83 (0.21)	48 (30.2)	39 (81.2)	0.77 (0.17)
Unvaccinated/doesn’t want to	17 (9.0)	10 (58.8)	0.83 (0.15)	14 (8.8)	11 (78.6)	0.78 (0.18)
p-value		0.77	0.57		0.52	0.97

HRQoL: health-related quality of life; SD: standard deviation; CNCD: chronic non-communicable diseases.

^a^ HRQoL - no problems = 1.000; any problem < 1.000.

^b^ Missing information for 3 individuals.

^c^ Having at least one factor considering insufficient physical activity, excessive alcohol consumption. smoking or regular consumption of unhealthy foods.

When the EQ-5D-3L domains were evaluated, there was a higher frequency of anxiety/depression problems at 6 months (44.9% *versus* 18.2%), problems with self-care at 12 months (22.9% *versus* 8.0%) and personal activities at 12 months (56.9% *versus* 22.0%) among patients with post-covid syndrome as compared to those without this condition (Table 4).

**Table 4:** HRQoL domainsa according to post-COVID-19 syndrome 6 and 12 months after hospital discharge due to COVID-19. Cuiabá (MT). 2024.

Domains	6 months (n = 189)					12 months (n = 159)				
	General		Post-COVID-19 syndrome^b^		p-value	Overall		Post-COVID-19^b^ syndrome		p-value
	n	%	No n (%)	Yes n (%)		n	%	No n (%)	Yes n (%)	
Mobility					< 0.01					0.23
No problem	155	82.1	22 (100.0)	133 (79.6)		121	76.1	41 (82.0)	80 (73.4)	
Some problem	34	17.9	0 (0.0)	34 (20.4)		38	23.9	9 (18.0)	29 (26.6)	
Self-care					0.78					0.02
No problem	178	94.2	21 (95.4)	157 (94.0)		130	81.8	46 (92.0)	84 (77.1)	
Some problem	11	5.8	1 (4.5)	10 (6.0)		29	18.2	4 (8.0)	25 (22.9)	
Usual activities					0.14					< 0.01
No problem	161	85.2	21 (95.4)	140 (83.8)		86	54.1	39 (78.0)	47 (43.1)	
Some problem	28	14.8	1 (4.5)	27 (16.2)		73	45.9	11 (22.0)	62 (56.9)	
Pain/illness					0.06					0.72
No problem	131	69.3	19 (86.4)	112 (67.1)		86	54.1	26 (52.0)	60 (55.1)	
Some problem	58	30.7	3 (13.6)	55 (32.9)		73	45.9	24 (48.0)	49 (44.9)	
Anxiety/depression					0.01					0.66
No problem	110	58.2	18 (81.8)	92 (55.1)		101	63.5	33 (66.0)	68 (62.4)	
Some problem	79	41.8	4(18.2)	75 (44.9)		58	36.5	17 (34.0)	41 (37.6)	

HRQoL: health-related quality of life.

^a^ HRQoL - no problems = 1.000; any problem < 1.000.

^b^ Post-COVID-19 syndrome classified as the presence of any persistent symptoms 12 weeks after hospital discharge.

In the adjusted model, post-COVID-19 syndrome remained associated with having any problem in HRQoL (RR = 2.43; 95%CI: 1.06–5.57) and the anxiety/depression domain (RR = 2.74; 95%CI: 1.08–7.01) at 6 months after discharge; these associations were not significant when assessing the outcomes at 12 months after hospital discharge (Table 5).

**Table 5:** Poisson regression of the association of the health-related quality of life score (EQ-5D-3L) at 6 and 12 months after discharge with post-COVID-19 syndrome. Cuiabá (MT). 2024.

Domains	Post-covid syndrome (ref: no)	6 months				12 months			
		Not adjusted		Adjusted^b^		Not adjusted		Adjusted^c^	
		RR	(95%CI)	RR	(95%CI)	RR	(95%CI)	RR	(95%CI)
HRQoL^a^									
	Yes	2.60	(1.19–5.70)	2.43	(1.06–5.57)	1.40	(0.93–2.10)	1.34	(0.88–2.04)
Mobility									
	Yes	-	–	–	–	5.32	(0.77–36.9)	3.48	(0.46–26.40)
Self-care									
	Yes	1.32	(0.18–9.85)	0.29	(0.01–6.94)	1.94	(0.50–7.58)	1.41	(0.21–9.58)
Usual activities									
	Yes	3.55	(0.50–25.0)	0.78	(0.07–9.10)	1.95	(0.90–4.28)	1.53	(0.66–3.54)
Pain/unwellness									
	Yes	2.41	(0.82–7.09)	1.32	(0.38–4.57)	1.95	(0.90–4.27)	1.81	(0.81–4.03)
Anxiety/depression									
	Yes	2.47	(1.00–6.10)	2.74	(1.08–7.0)	0.78	(0.45–1.34)	1.15	(0.64–2.09)

HRQoL: health-related quality of life; 95%CI: 95% confidence interval; CNCD: chronic non-communicable diseases; RR: risk ratio.

^a^ HRQoL - no problems = 1.000; any problem < 1.000.

^b^ Adjusted for: gender, age group, education, race/color, number of comorbidities.

^c^ Adjusted for: sex, age group, schooling, race/color, number of comorbidities and co-occurrence of risk factors for NCDs.

## DISCUSSION

In addition to the high proportion of patients hospitalized for COVID-19 who had post-COVID-19 syndrome and impaired HRQoL 6 and 12 months after hospital discharge, the results of this study revealed an independent association between post-COVID-19 syndrome and worse HRQoL 6 months after discharge. When evaluating the domains of the EQ-5D scale, it was found that the presence of post-COVID-19 syndrome increased the risk of anxiety/depression by more than 2.7 times at 6 months after discharge.

The results of this study highlight the persistence of the impacts of COVID-19 on patients’ health-related quality of life after initial recovery. The high proportion of patients who reported at least one symptom of COVID-19 6 and 12 months after hospital discharge highlights the prolonged and debilitating nature of the post-COVID-19 syndrome, which continues to affect patients’ lives well beyond the acute phase of the infection^7,9,17^.

In addition, it has been found that the length of hospital stay and the severity of COVID-19 in the acute phase, such as the need for mechanical ventilation, are factors that have contributed to reduced HRQoL after recovery^19^. However, in this study, post-COVID-19 syndrome remained significantly associated with worse HRQoL even after adjusting for factors linked to worse severity of COVID-19, highlighting the lasting impact of this condition.

The proportion of individuals who reported some impairment in quality of life at 6 and 12 months was high. Notably, this proportion increased from 59.2% to 77.0% of the patients evaluated, respectively. These data are alarming and highlight the urgent need for public health strategies such as physical and cognitive rehabilitation centers, monitoring and development of protocols for tracking persistent symptoms, and integration and training of primary care to identify cases of post-COVID-19 syndrome.

Post-COVID-19 syndrome and HRQoL were not associated at 12 months after discharge in this study. However, studies conducted in the USA^19^after 24 months, in Brazil^8^after 12 months and in China^15^after 24 months from hospital discharge found that persistent symptoms were associated with a decrease in quality of life during follow-up.

In the USA^19^, after 24 months, persistent symptoms were associated with a decrease in quality of life, with significant variability depending on such factors as age, comorbidities, and severity of infection^19^. In China^15^, symptoms such as anxiety and depression had an impact on quality of life, showing that the effects of COVID-19 go beyond the acute period^15^.

The Brazilian study by Ida et al.^8^used the SF-36 to assess quality of life, while this study applied the EQ-5D-3L by domains, which may specify differences in the findings. The authors found that 67% of inpatients had problems with motor function, and a third of them had not returned to work after 12 months. Additionally, significant emotional changes were identified, corroborating our results^8^.

The literature that has considered post-COVID-19 syndrome at different points in time has found both positive associations with quality of life^19,22,27^ and no association^28^.

In a longitudinal study with 24 months of follow-up^19^, it was observed that the symptoms of post-COVID-19 syndrome reach their peak and quality of life reaches its lowest point between 6 and 12 months after infection, which is in line with the findings of this study. In addition, the proportion of patients reporting HRQoL problems increased between the 6- and 12-month assessments, confirming the trend observed by Demko et al.^19^that the impact on quality of life is more pronounced during this period, due to residual inflammation and biological factors of the disease that can worsen and exacerbate persistent symptoms. The literature^11,17,19^has shown that the presence of post-COVID-19 syndrome is associated with decreased HRQoL in domains such as physical functioning and general health. This study showed that persistent symptoms at 6 months were associated with the anxiety/depression domain. These findings are consistent with the literature indicating that persistent symptoms have been associated with higher levels of psychological distress and stress^17,19,27^.

The effects of COVID-19 on the nervous system are believed to be linked to the angiotensin-converting enzyme receptor 2-ECA^2^, which is present in neurons and the choroid plexus, albeit in low quantities. The neurophysiology of these cognitive symptoms is not well understood, however, biological factors, such as the effects of chronic inflammation generated by the infection, and psychological factors, including fear and the impact of social isolation, can interfere with essential brain functions and decrease quality of life^6,29^. Moreover, social support is an independent predictor of lower feelings of anxiety, lower risk of depression and better perceived quality of life in COVID-19 patients^29^.

The prospective study^19^highlights that pre-COVID-19 diagnoses of anxiety and depression were not associated with post-COVID-19 syndrome, however those with these diagnoses at the beginning of the study had a consistently worse quality of life than those without these diagnoses. The findings in the literature^16,22,29,30^, which reveal a high prevalence of psychiatric and cognitive impairments, including common mental disorders, depression and anxiety 6 to 9 months after SARS-CoV-2 infection, corroborate our results, which also identified impacts on mental health 6 months after COVID-19 infection, which may be related to post-COVID-19 syndrome and result in impaired quality of life.

This study followed up the same individuals at two points in time, at 6 and 12 months, using a validated questionnaire to assess HRQoL^21^. The follow-up rate at 6 months (73.3%) and 12 months (62.5%) can be considered high as compared to other Brazilian studies^8,18^ which had low follow-up percentages (38.0% to 56.0%). In addition, this study sought to analyze the independent association of post-COVID-19 syndrome with HRQoL using an adjustment model based on the causal diagram, which contributes to understanding this relationship, since no studies have been found in the scientific literature that consider adjustments for potential confounding factors.

The limitations of this study include convenience sampling, which prevents the findings from being generalized. The absence of information such as risk factors for NCDs before COVID-19 infection and at 6 months also limited the assessment of these factors, such as previous exposure to quality of life assessment. Another limitation of this study is the fact that we did not assess the HRQoL of individuals before hospitalization, in order to assess its change after hospitalization for COVID-19 and the association with post-COVID-19 syndrome. Furthermore, we cannot ignore the possible memory bias for some retrospective questions and about the symptoms present in the acute phase during hospital admission.

In conclusion, our results suggest that patients with post-COVID-19 syndrome have impaired HRQoL up to six months after hospital discharge and are more likely to have anxiety/depression six months after hospital discharge. These results reveal the long-term impacts on the physical and emotional functions and mental health of those affected. There is a need for comprehensive primary care action on the long-term effects and their impact on HRQoL, with psychological support strategies and personalized interventions that can improve the quality of life of these individuals and prevent a worsening of mental and physical symptoms over time.

## Data Availability

The datasets generated and/or analyzed during this study are not publicly available due to [ethical/legal/privacy] restrictions.
