# Supplementary material for: Post-COVID-19 syndrome and health-related quality of life after hospital discharge
Source: Rev Saude Publica. 2025 Dec 1;59:e34. doi: 10.11606/s1518-8787.2025059006650 (PMC12673641; doi:10.11606/s1518-8787.2025059006650)
Supplement: Translation

# Translation: Síndrome pós-covid-19 e qualidade de vida relacionada à saúde após alta hospitalar

**Authors:** Roseany Patricia Silva Rocha, Amanda Cristina de Souza Andrade, Ana Paula Muraro

**Affiliations:** I Universidade Federal de Mato Grosso. Instituto de Saúde Coletiva. Cuiabá, MT, Brasil

**Keywords:** Síndrome Pós-Covid-19, Hospital, Qualidade de vida

## Abstract

**OBJETIVO:**

Analisar a associação entre presença de síndrome pós-covid-19 após 6 meses e a qualidade de vida relacionada à saúde após 6 e 12 meses da alta hospitalar.

**MÉTODOS:**

Estudo de coorte ambidirecional, realizado com indivíduos que receberam alta em três dos principais hospitais de Cuiabá (MT) entre outubro de 2021 e março de 2022. Após coleta de dados em prontuários, os indivíduos foram entrevistados por telefone após 6 meses (n = 189) e 12 meses (n = 159) da alta hospitalar, sendo avaliada a presença de pelo menos um sintoma persistente pós-covid-19 aos 6 meses (síndrome pós-covid-19) e a qualidade de vida relacionada à saúde (EQ-5D-3L) aos 6 e 12 meses, bem como das dimensões específicas (mobilidade, autocuidado, atividades habituais, dor/desconforto e ansiedade/depressão). A associação foi avaliada pela regressão de Poisson com variância robusta, ajustada para características sociodemográficas, de saúde e internação.

**RESULTADOS:**

Dos indivíduos avaliados, 88,4% responderam que apresentaram sintomas da covid-19 na entrevista após 6 meses. Aos 6 e 12 meses de alta hospitalar, 55,0% e 74,2% dos indivíduos, respectivamente, apresentaram algum prejuízo na qualidade de vida. No modelo múltiplo, a síndrome pós-covid-19 manteve-se associada a ter qualquer problema de qualidade de vida (RR = 2,43; IC95% 1,06–5,57) e especificamente ao domínio ansiedade/depressão (RR = 2,74; IC95% 1,08–7,01) aos 6 meses após a alta. A associação não se manteve significativa após 12 meses.

**CONCLUSÃO:**

Esses resultados demonstram as repercussões negativas a longo prazo da síndrome pós-covid-19 nas funções cognitivas, emocionais e físicas, expondo o impacto negativo para a qualidade de vida das pessoas acometidas.

## INTRODUÇÃO

A pandemia de covid-19, declarada pela Organização Mundial da Saúde (OMS) em março de 2020, representou uma grave ameaça à saúde pública global, com mais de 712 mil mortes no Brasil registradas pela doença^1^. Com o avanço de novos estudos, ficou evidente que o SARS-CoV-2 não causa apenas problemas respiratórios, mas pode desencadear uma síndrome que afeta múltiplos órgãos. Além da infecção aguda inicial, foram identificados diversos sintomas duradouros e novos da covid-19^4^.

Após mais de um ano do início dessa crise, a OMS anunciou oficialmente, em outubro de 2021, a existência da “síndrome pós-covid-19” como uma condição clínica específica^5^, descrevendo um conjunto diversificado de sintomas que persistem por mais de 12 semanas após a infecção, sem outra explicação diagnóstica, sendo os sintomas mais frequentes fadiga, dispneia, dor nas articulações, perda de memória, perda de cabelo e ansiedade^6^. A OMS apresentou uma definição clínica oficial, considerando a nomenclatura de “condição pós-covid-19”^4,5^ e o Centro de Controle e Prevenção de Doenças dos Estados Unidos por “condições pós-covid”, ou *post-covid conditions* (PCC) em inglês^10^. Outras nomenclaturas também são encontradas na literatura científica, como “covid longa”, “covid-19 pós-aguda”, “efeitos em longo prazo da covid”, “síndrome covid pós-aguda,” “síndrome de covid crônica” entre outras denominações^3,11^. O Ministério da Saúde (MS) optou pelo termo “condições pós-covid” para padronizar documentos técnicos e orientar profissionais de saúde sobre o tema^12^.

A proporção de indivíduos que apresentam síndrome pós-covid-19 após recuperação da fase aguda da covid-19 tem variado entre os estudos^7,9,11^. Revisão sistemática e meta-análise com 41 estudos meta-analisados de 16 países, principalmente China e Estados Unidos, com pacientes adultos com mais de seis meses de acompanhamento identificou a prevalência global de 43,0% de condições pós-covid, sendo maior entre os pacientes que necessitaram de internação (54,0%) quando comparados aos não hospitalizados (34,0%). A fadiga foi o sintoma mais relatado seguido de problemas de memória. Apenas um estudo brasileiro foi incluído na revisão, com pacientes internados e média de 6 meses de acompanhamento, sendo avaliado especificamente sintomas cognitivos e psiquiátricos^11^.

A qualidade de vida relacionada à saúde (QVRS) é definida como a avaliação subjetiva que um indivíduo faz do impacto de sua saúde em diferentes aspectos da sua vida. É uma medida do bem-estar físico, psicológico e social, influenciada por experiências, crenças, expectativas e percepções individuais^13^.

No contexto dinâmico da pandemia, a QVRS tem despertado grande interesse da comunidade científica, especialmente no que se refere ao impacto da doença na função física e cognitiva dos pacientes e na satisfação geral com a vida^14^. Estudos recentes têm avaliado a qualidade de vida entre adultos com sintomas persistentes, indicando significativa redução na qualidade de vida, com destaque para o aumento de sintomas de ansiedade, depressão e dificuldades cognitivas^8,17^.

Uma revisão sistemática e meta-análise de estudos com tempo de acompanhamento variando de um a seis meses identificou que 59% dos pacientes com sintomas persistentes relataram baixa qualidade de vida^17^. Os sintomas persistentes mais prevalentes foram fadiga, dispneia, anosmia, artralgia, dor de cabeça e distúrbios do sono, além de impactos na saúde mental. A análise de meta-regressão demonstrou que a baixa qualidade de vida foi significativamente maior entre pacientes que necessitaram de admissão na UTI durante a infecção aguda^17^.

No contexto nacional, resultados encontrados por Ida et al.^8^ em pacientes adultos com idade entre 40 a 59 anos indicam que aqueles com fadiga, artralgia e dispneia persistente por mais de 12 meses apresentaram pior qualidade de vida em comparação com aqueles que não tinham sintomas persistentes da covid-19.

Outro ponto crucial é que, apesar do aumento no número de evidências sobre os possíveis impactos da covid-19 na qualidade de vida, a relação entre a síndrome pós-covid-19 e a QVRS ainda não está completamente elucidada. Muitos estudos existentes não consideraram ajustes para potenciais fatores de confusão, como características sociodemográficas, condições clínicas preexistentes e variáveis relacionadas à internação^8,15,19^. A falta de ajustes pode comprometer a validade dos achados e gerar interpretações enviesadas sobre a associação entre a síndrome e a QVRS. Nesse sentido, este estudo busca preencher essa lacuna, oferecendo uma análise mais robusta e precisa sobre o tema.

Diante disso, este estudo propõe analisar a associação entre a presença de síndrome pós-covid-19 após 6 meses e a qualidade de vida relacionada à saúde após 6 e 12 meses da alta hospitalar, ajustando para variáveis sociodemográficas e características relacionadas à internação.

## MÉTODOS

### População de Estudo

Trata-se de um estudo de coorte ambidirecional, realizado com pacientes confirmados com covid-19, residentes em Cuiabá e Várzea Grande (que compõe a região metropolitana do estado), que foram internados em hospitais públicos e privados em Cuiabá e com fechamento do caso como alta hospitalar entre outubro e 2021 e março de 2022.

Os casos foram identificados pelo IndicaSUS, sistema implantado pelo governo de Mato Grosso^20^, por meio do acesso junto à Secretaria Municipal de Saúde de Cuiabá. Após coleta de dados em prontuários, os indivíduos foram entrevistados por telefone 6 e 12 meses após a alta hospitalar.

Dos sete hospitais de Cuiabá, apenas três permitiram o acesso aos prontuários dos pacientes. Foram estabelecidos como elegíveis os indivíduos de 18 anos ou mais de idade, casos confirmados (por meio do exame de reação em cadeia da polimerase [PCR], teste rápido ou teste de antígeno, ou, ainda, confirmação por exames de imagem) e que receberam alta hospitalar segundo informações registradas no IndicaSUS. Estabeleceu-se como critérios de exclusão: pacientes que residem em casa de repouso e pacientes que tiverem dificuldades de comunicação (afasia, comprometimento cognitivo e grave perda auditiva).

Foram identificados 998 casos confirmados de covid-19 de residentes de Cuiabá e Várzea Grande que tiveram fechamento do caso nos hospitais da capital no período considerado. Destes, 689 receberam alta hospitalar (69,0%), sendo 277 nos três hospitais que permitiram o acesso aos prontuários (40,2% do total dos pacientes adultos que tiveram alta no período). Durante a coleta nos prontuários, 10 pacientes foram excluídos por residirem em casa de repouso e oito que, apesar de estarem registrados no IndicaSUS como alta hospitalar, foram a óbito durante a internação. Portanto, permaneceram elegíveis para o estudo 259 indivíduos, que tiveram os dados coletados dos prontuários, fase retrospectiva da coorte. O detalhamento da seguimento da população de estudo pode ser acessada na publicação de Rocha et al.^9^ Para esta análise, foram ainda excluídos três indivíduos devido à falta de muitas informações no prontuário, sendo analisados então 256 indivíduos.

### Coleta de Dados

As etapas prospectivas foram compostas por duas avaliações por entrevistas telefônicas. Aos 6 meses foram entrevistados 190 indivíduos (73,3% da linha de base) e 160 indivíduos foram entrevistados aos 12 meses. Foram identificados o total de 28 óbitos por meio do Sistema de Informação sobre Mortalidade, consultado pela Secretaria Municipal de Saúde de Cuiabá. Para a análise dos dados, houve a necessidade de excluir um paciente que não apresentava as respostas completas no questionário de qualidade de vida relacionados à saúde na entrevista após seis meses da alta hospitalar, portanto, considerou-se 189 pacientes aos seis meses e 159 aos 12 meses.

As entrevistas foram individuais, realizadas via telefone por meio de questionário estruturado com questões sobre características demográficas e socioeconômicas, moradia e condições de vida, características da saúde e dados de internação e sintomas persistentes desde a fase aguda da covid-19 ou novos até o momento da entrevista.

### Variáveis de Exposição e Desfecho

A variável desfecho deste estudo foi a QVRS, avaliada pela versão de três níveis do questionário EQ-5D (EQ-5D-3L) validado para avaliação da qualidade de vida relacionada à saúde na população brasileira^21^, que compreende cinco domínios: mobilidade, autocuidado, atividades habituais, dor/desconforto e ansiedade/depressão. O participante descreveu os níveis de gravidade de cada dimensão em uma escala de 1 a 3, em que 1 indica nenhum problema, 2 indica algum problema e 3 indica problema extremo. Portanto, são possíveis 243 combinações diferentes de estados de saúde.

Cada combinação de respostas nas cinco dimensões gera um perfil de estado de saúde de cinco dígitos, como 11111 (nenhum problema em qualquer dimensão) ou 22332 (alguns problemas em mobilidade, autocuidado, atividades usuais etc.). A combinação pode ser traduzida em um único valor de índice resumido que varia de −0,1755 (pior que morto) a 1,000 (QVRS perfeita) e é ponderado de acordo com os valores e preferências brasileiros em relação aos resultados de saúde^21^. O escore do EQ-5D-3L foi avaliado de forma contínua e categórica, sendo para essa última adotada a dicotomização em 1,0 para nenhum problema e menor que 1,0 para qualquer problema de QVRS^22^. Os domínios foram organizados em: mobilidade, cuidados pessoais, atividades comuns, dor/mal-estar, ansiedade e depressão. As respostas para cada domínio foram definidas como “sem problema” ou “com algum problema”, de acordo com a pontuação previamente descrita.

A variável de exposição de interesse foi a síndrome pós-covid-19, considerada como a presença de sintomas persistentes no momento da entrevista aos 6 e 12 meses de alta hospitalar^7^. Foram questionados sobre 24 classes de sintomas presentes na fase aguda de covid-19 e após 6 e 12 meses de alta hospitalar, essa lista de sintomas foi construída a partir da literatura^23^. Os sintomas foram classificados em muscular, neuropsiquiátrico, dermatológico, cardiovascular e pulmonar e estão detalhados no artigo de Rocha et al.^9^

### Variáveis de Ajuste

As variáveis sociodemográficas e econômicas analisadas foram: sexo (feminino; masculino), faixa etária (18 a 29 anos; 30 a 49 anos; 50 a 59 anos; 60 anos ou mais), raça/cor (branca; parda; preta; amarela/indígena; e ignorado), escolaridade (fundamental completo; ensino médio completo; superior completo ou mais), renda mensal (considerando o salário mínimo vigente quando realizada a coleta de dados R$ 1.212,00) *per capita* (até 2 salários; 2 a 3 salários mínimos; 3 ou mais salários mínimos), situação de trabalho atual aos seis meses (sim; não), moradia (regular; bom/ muito bom). Em relação às características de saúde e internação, considerou-se o autorrelato do número de doses de vacinas recebidas antes da internação (1; 2; 3 ou 4 doses), e as variáveis obtidas por meio do acesso ao prontuário hospitalar foram: tempo de internação (dias de internação avaliada em média e classificada em tercil; primeiro tercil de 1 a 6 dias, segundo tercil de 7 a 12 dias, terceiro tercil de 13 a 134 dias), internação em unidade de tratamento intensivo (UTI) (sim; não), necessidade de ventilação mecânica (sim; não).

Para avaliar o número de comorbidades, foi considerado o autorrelato de diagnóstico de hipertensão arterial, diabetes mellitus e doença de coração, asma/bronquite, câncer, doença renal, doença pulmonar, obesidade, transtorno mental ou depressão ou outras doenças crônicas. Para a análise das comorbidades individualmente, considerou-se hipertensão arterial, diabetes e doença do coração, por terem sido as mais frequentes entre as comorbidades. Para classificar a obesidade, foi considerado o peso e estatura autorrelatado nas entrevistas realizadas após 6 meses da alta hospitalar para o cálculo do índice de massa corporal, apenas três indivíduos não souberam informar e permaneceram como sem informação. A obesidade foi classificada como com 30kg/m^2^ ou mais^24^. Além disso, considerou-se também a coocorrência de hipertensão arterial, diabetes mellitus, doença de coração e obesidade, classificadas em: sem comorbidades, uma ou duas comorbidades e três ou mais comorbidades.

Foi considerada variável de coocorrência de fatores de risco para doenças crônicas não transmissíveis (DCNT), avaliadas 12 meses após a alta hospitalar, composta pela combinação de: tabagismo, consumo excessivo de álcool, atividade física insuficiente e consumo regular de alimentos não saudáveis. Foi considerado como exposição ao tabagismo o indivíduo que relatou ser fumante atual ou ex-fumante, consumo excessivo de bebida alcóolica os que relataram consumo maior de cinco doses para homens e quatro para mulheres em uma única ocasião^25^. Atividade física foi avaliada utilizando o instrumento Questionário Internacional de Atividade Física (IPAQ) versão curta, sendo considerado como insuficiente a prática inferior a 150 minutos por semana^26^. Foi considerada como inadequado a presença de pelo menos um fator de risco para DCNT.

### Análise de Dados

As variáveis incluídas no modelo ajustado foram identificadas por meio de gráfico acíclico direcionado (DAG) com o auxílio do programa Daggity, versão 3.016, identificado o conjunto mínimo de variáveis de ajuste a serem utilizadas em modelos múltiplos para estimativa da associação entre estas duas variáveis. O ajuste mínimo identificado incluiu: sexo, faixa etária, escolaridade, raça/cor, tempo de internação, internação em UTI, número de comorbidades e coocorrência de fatores de risco para DCNT (Figura).

**Figura:** Gráfico acíclico direcionado representando hipóteses sobre as relações entre exposição, desfecho e demais covariáveis a condição pós-covid-19. Cuiabá (MT), 2024.

UTI: unidade de terapia intensiva; QVRS: qualidade de vida relacionado à saúde; DCNT: doenças crônicas não transmissíveis.

Nota: comorbidades (sem comorbidades, 1 ou 2 comorbidades e 3 ou mais comorbidades). Para análise foi adotado o termo coocorrência de fatores de risco para DCNT para substituir estilo de vida.

As análises foram realizadas utilizando o software Stata, versão 16.0. Foi estimada a taxa de seguimento e comparada a distribuição das variáveis sociodemográficas entre os participantes e não participantes com o objetivo de verificar possíveis perdas seletivas. Foi realizada análise descritiva com cálculo de frequências absolutas e relativas para as variáveis categóricas e cálculo da média e desvio-padrão (DP) para as variáveis quantitativas. Para a comparação do QVRS contínua conforme as variáveis explicativas foram realizados o teste de *t* para amostras independentes (comparação de dois grupos) ou Anova (comparação de três grupos ou mais), se satisfeita a suposição de normalidade. Caso, contrário, foram utilizados os testes não paramétricos de Mann-Whitney e Kruskall Wallis, respectivamente. Para avaliar a associação entre QVRS dicotomizada e as variáveis explicativas foi utilizado o teste qui-quadrado de Pearson. Para a análise múltipla foram estimadas as razões de risco (RR) e respectivos p-valor e intervalo de confiança de 95% (IC95%) por meio de regressão de Poisson com variância robusta para avaliar a associação da variável independente de interesse (síndrome pós-covid-19) e o desfecho (qualidade de vida e seus domínios).

### Considerações Éticas

Este estudo foi aprovado pelo Comitê de Ética em Pesquisa com Seres Humanos da Área da Saúde da Universidade Federal de Mato Grosso-Campus Cuiabá (Parecer n. 5.415.255/2022, de 18 de maio de 2022). Todos os participantes assinaram o termo de consentimento livre e esclarecido.

## RESULTADOS

Foram avaliados 189 (73,0% dos elegíveis) pacientes após 6 meses da alta hospitalar (média: 6,29 meses, DP = 1,24) e 159 (61,4% dos elegíveis) pacientes após 12 meses da alta hospitalar (média: 13,17 meses, DP = 1,11). Aos 6 meses da alta hospitalar, 55,0% dos indivíduos foram classificados como com qualquer problema de QVRS. Essa proporção foi de 74,2% após 12 meses da alta (dados não mostrados em tabela).

A taxa de seguimento manteve-se menor entre os homens (64,1% e 53,3%) em relação às mulheres (82,3% e 69,8%) com idade de 60 anos ou mais (64,4% e 43,2%) quando comparados as faixas etárias mais novas e entre as de raça/cor preta (53,3% e 46,6%) após 6 e 12 meses da alta hospitalar, respectivamente. Não houve diferença significativa na taxa segundo raça/cor, tempo de internação e se internado em UTI (Tabela 1).

**Tabela 1:** Características sociodemográficas e de internação e taxa de seguimento 6 e 12 meses. Cuiabá (MT), 2024.

	Linha de base (n = 256)	6 meses (n = 89)		12 meses (n = 159)	
	n (%)	n (%)	Taxa de seguimento (%)	n (%)	Taxa de seguimento (%)
Sexo					
Masculino	120 (46,8)	77 (40,7)	64,1	64 (40,2)	53,3
Feminino	136 (53,2)	112 (59,3)	82,3	95 (59,7)	69,8
p-valor		< 0,01		0,02	
Faixa etária					
18 a 29 anos	29 (11,3)	23 (12,1)	79,3	20 (12,5)	68,9
30 a 49 anos	74 (28,9)	61 (32,2)	82,4	59 (37,1)	79,7
50 a 59 anos	35 (13,6)	29 (15,3)	82,8	29 (18,2)	82,8
≥ 60 anos	118 (46,1)	76 (40,2)	64,4	51 (32,1)	43,2
p-valor		0,01		< 0,01	
Raça/cor					
Branca	42 (16,4)	36 (19,0)	85,7	34 (21,4)	80,9
Parda	166 (64,8)	120 (63,5)	72,3	96 (60,4)	57,8
Preta	15 (5,8)	8 (4,2)	53,3	7 (4,4)	46,6
Amarela	15 (5,8)	11 (5,8)	73,3	10 (6,3)	66,6
Ignorado	18 (7,0)	14 (7,4)	77,7	12 (7,5)	66,6
p-valor		0,15		0,30	
Tempo de internação (dias) tercil					
1º tercil (1 a 6 dias)	91 (35,5)	73 (38,8)	80,2	64 (40,2)	70,3
2º tercil (7 a 12 dias)	88 (34,5)	61 (32,4)	69,3	49 (30,0)	55,7
3º tercil (13 a 134 dias)	77 (30,2)	54 (28,7)	70,1	46 (29,1)	59,7
p-valor		0,14		0,61	
Internado em UTI					
Sim	77 (30,1)	57 (30,1)	74,0	45 (28,3)	58,4
Não	179 (69,9)	132 (69,8)	73,7	114 (71,7)	63,6
p-valor		0,96		0,20	

UTI: unidade de terapia intensiva.

Após 6 meses da alta hospitalar, 88,4% dos indivíduos foram classificados com síndrome pós-covid-19. Foi maior a proporção de indivíduos que relataram qualquer problema de qualidade de vida aos 6 e 12 meses da alta hospitalar (59,3% e 77,0%) entre aqueles que apresentavam síndrome pós-covid-19 aos 6 meses, quando comparados aos sem essa condição (22,7% e 55,0%, respectivamente), com diferença significativa (p-valor < 0,01 e p-valor = 0,03, respectivamente). Foi significativamente maior a média e a proporção de qualquer problema de QVRS aos 6 meses entre os indivíduos de menor renda mensal e que necessitaram de ventilação mecânica durante a internação; e aos 12 meses entre aqueles de menor escolaridade, as demais variáveis não mostraram diferença significativa (Tabela 2).

**Tabela 2:** Média do escore e proporção de qualquer problema de QVRS, segundo fatores sociodemográficos e relacionados à internação 6 e 12 meses após a alta hospitalar. Cuiabá (MT), 2024.

Variáveis	6 meses (n = 189)			12 meses (n = 159)		
	Geral	QVRS^a^		Geral	QVRS^a^	
	n (%)	Qualquer problema^a^ n (%)	Média (DP)	n (%)	Qualquer problema^a^ n (%)	Média (DP)
Síndrome pós-covid-19 aos 6 meses^b^						
Sim	167 (88,4)	99 (59,3)	0,84 (0,16)	139 (87,4)	107 (77,0)	0,84 (0,19)
Não	22 (11,6)	5 (22,7)	0,91 (0,22)	20 (12,6)	11 (55,0)	0,77 (0,19)
p-valor		< 0,01	0,08		0,03	< 0,01
Sexo						
Masculino	77 (41,0)	38 (49,3)	0,86 (0,19)	64 (40,2)	44 (68,7)	0,79 (0,20)
Feminino	112 (59,0)	66 (58,9)	0,84 (0,16)	95 (59,7)	74 (77,9)	0,77 (0,18)
p-valor		0,44	0,39		0,12	0,41
Faixa etária na internação						
18 a 29 anos	23 (12,1)	13 (56,5)	0,89 (0,23)	20 (12,6)	16 (80,0)	0,80 (0,14)
30 a 49 anos	61 (32,3)	31 (50,8)	0,86 (0,23)	59 (37,1)	38 (64,4)	0,80 (0,18)
50 a 59 anos	29 (15,3)	18 (62,1)	0,84 (0,26)	29 (18,2)	20 (68,9)	0,78 (0,21)
≥ 60 anos	76 (40,0)	42 (55,2)	0,84 (0,21)	51 (32,1)	44 (86,3)	0,74 (0,20)
p-valor		0,62	0,64		0,29	0,34
Raça/cor						
Branca	36 (18,9)	20 (55,5)	0,84 (0,18)	34 (21,4)	25 (73,5)	0,76 (0,22)
Parda	120 (63,2)	66 (55,0)	0,85 (0,17)	96 (60,4)	69 (71,9)	0,78 (0,19)
Preta	8 (4,2)	4 (50,0)	0,86 (0,15)	7 (4,4)	5 (71,4)	0,86 (0,09)
Amarela	11 (5,8)	6 (54,5)	0,83 (0,16)	10 (6,3)	10 (100)	0,72 (0,11)
Ignorado	14 (7,4)	8 (57,1)	0,86 (0,13)	12 (7,5)	9 (75,0)	0,76 (0,19)
p-valor		0,57	0,97		0,69	0,64
Escolaridade						
Até fundamental completo	106 (55,8)	58 (54,7)	0,84 (0,17)	80 (50,3)	69 (86,2)	0,73 (0,20)
Ensino médio completo	51 (26,8)	25 (49,0)	0,87 (0,15)	49 (30,8)	30 (61,2)	0,82 (0,18)
Superior completo ou mais	32 (16,9)	21 (65,6)	0,83 (0,19)	30 (18,8)	19 (63,3)	0,83 (0,14)
p-valor		0,39	0,40		< 0,01	< 0,01
Renda mensal ao 6 meses^c^						
Até 2 SM	59 (32,1)	39 (66,1)	0,81 (0,17)	48 (31,2)	43 (89,6)	0,75 (0,14)
2 a 3 SM	58 (30,6)	26 (44,8)	0,89 (0,14)	45 (28,3)	29 (64,4)	0,79 (0,21)
≥ 3 SM	66 (35,8)	34 (51,5)	0,87 (0,13)	60 (37,7)	41 (68,3)	0,19 (0,21)
p-valor		0,03	0,03		0,12	0,49
Moradia						
Regular	10 (5,3)	6 (60,0)	0,80 (0,22)	10 (100,0)	8 (80,0)	0,07 (0,18)
Bom/ muito bom	179 (94,7)	98 (54,7)	0,85 (0,17)	149 (83,2)	110 (73,8)	0,78 (0,19)
p-valor		0,49	0,39		0,66	0,63
Situação de trabalho atual aos 6 meses						
Sim	78 (41,2)	34 (43,6)	0,89 (0,13)	73 (93,6)	46 (63,0)	0,81 (0,18)
Não	111 (58,7)	70 (63,1)	0,82 (0,18)	86 (77,5)	72 (83,7)	0,75 (0,19)
p-valor		< 0,01	0,04		< 0,01	0,02
Tempo de internação (dias) - tercil						
1º tercil (1 a 6 dias)	74 (39,1)	41 (55,4)	0,86 (0,16)	64 (40,2)	44 (69,8)	0,78 (0,20)
2º tercil (7 a 12 dias)	61 (32,4)	31 (50,8)	0,85 (0,17)	49 (30,8)	39 (79,6)	0,75 (0,20)
3º tercil (13 a 134 dias)	54 (28,7)	32 (59,2)	0,83 (0,18)	46 (28,9)	35 (76,1)	0,79 (0,16)
p-valor		0,12	0,63		0,14	0,51
Necessitou de internação em UTI						
Sim	57 (30,0)	32 (56,1)	0,83 (0,20)	45 (28,3)	34 (75,5)	0,79 (0,18)
Não	132 (69,8)	72 (54,5)	0,86 (0,16)	114 (71,7)	84 (73,7)	0,77 (0,19)
p-valor		0,54	0,35		0,29	0,56
Necessitou de ventilação mecânica						
Sim	9 (4,8)	7 (77,8)	0,07 (0,15)	8 (5,0)	7 (87,5)	0,76 (0,21)
Não	180 (95,2)	97 (53,9)	0,89 (0,12)	151 (95,0)	111 (73,5)	0,79 (0,19)
p-valor		< 0,01	< 0,01		0,83	0,69

QVRS: qualidade de vida relacionado à saúde; DP: desvio padrão; SM: salário mínimo; UTI: unidade de terapia intensiva.

^a^ QVRS- sem problemas = 1,000; qualquer problema < 1,000.

^b^ Síndrome pós-covid-19 classificada como a presença de algum sintoma persistente 6 meses alta hospitalar.

^c^ Foi considerado o salário vigente R$ 1.212,00. Salário mínimo apenas aos 6 meses de alta hospitalar.

Aos seis meses da alta hospitalar, verificou-se menor média de QVRS e maior proporção de qualquer problema de QVRS entre os indivíduos com hipertensão, doença do coração e com duas ou mais comorbidades quando internado. Entretanto, essas diferenças não se mantiveram significativas quando avaliadas aos 12 meses da alta hospitalar (Tabela 3).

**Tabela 3:** Média do escore e proporção de qualquer problema de QVRS segundo condições de saúde 6 e 12 meses após a alta hospitalar. Cuiabá (MT), 2024.

Variáveis	6 meses (n = 189)			12 meses (n = 159)		
	Geral	QVRS^a^		Geral	QVRS^a^	
	n (%)	Qualquer problema^a^ n (%)	Média (DP)	n (%)	Qualquer problema^a^ n (%)	Média (DP)
Comorbidades						
Hipertensão arterial						
Não	78 (49,0)	39 (50,0)	0,88 (0,15)	72 (45,2)	49 (68,1)	0,79 (0,18)
Sim	111 (69,8)	65 (58,5)	0,83 (0,18)	87 (54,7)	69 (79,3)	0,76 (0,19)
p-valor		0,24	0,02		0,10	0,23
Diabetes mellitus						
Não	125 (66,1)	66 (52,8)	0,87 (0,15)	111 (69,8)	80 (72,1)	0,78 (0,19)
Sim	64 (33,8)	38 (59,4)	0,82 (0,19)	48 (30,2)	38 (79,2)	0,77 (0,17)
p-valor		0,39	0,06		0,67	0,87
Obesidade^b^						
Não	179 (94,7)	97 (54,2)	0,86 (0,18)	151 (94,9)	113 (74,8)	0,79 (0,17)
Sim	10 (20,7)	7 (70,0)	0,83 (0,16)	8 (5,0)	5 (62,5)	0,76 (0,23)
p-valor		0,32	0,32		0,43	0,35
Doença do coração						
Não	181 (95,7)	96 (53,0)	0,86 (0,16)	151 (94,9)	110 (72,8)	0,78 (0,19)
Sim	8 (4,2)	8 (100,0)	0,63 (0,22)	8 (5,0)	8 (100,0)	0,73 (0,12)
p-valor		< 0,01	< 0,01		0,30	0,47
Número de comorbidades na internação						
Sem comorbidades	53 (27,9)	24 (45,3)	0,89 (0,16)	50 (31,4)	34 (68,0)	0,79 (0,20)
1 ou 2	124 (65,6)	70 (56,4)	0,84 (0,16)	98 (61,6)	73 (74,5)	0,78 (0,17)
3 ou mais	12 (6,3)	10 (83,3)	0,72 (0,22)	11 (6,9)	11 (100,0)	0,64 (0,26)
p-valor		< 0,01	< 0,01		0,08	0,04
Coocorrência de fatores de risco para DCNT^c^						
Não	-	-	-	22 (13,8)	14 (63,6)	0,79 (0,22)
Sim	-	-	-	137 (86,1)	104 (75,9)	0,77 (0,18)
p-valor					0,22	< 0,32
Vacinação						
1 dose	32 (16,9)	19 (59,4)	0,87 (0,11)	30 (18,8)	21 (70,0)	0,79 (0,20)
2 doses	78 (41,2)	40 (51,3)	0,86 (0,16)	67 (42,1)	47 (70,1)	0,78 (0,20)
3 ou 4 doses	62 (32,8)	35 (56,4)	0,83 (0,21)	48 (30,2)	39 (81,2)	0,77 (0,17)
Não vacinado/não pretende	17 (9,0)	10 (58,8)	0,83 (0,15)	14 (8,8)	11 (78,6)	0,78 (0,18)
p-valor		0,77	0,57		0,52	0,97

QVRS: qualidade de vida relacionado à saúde; DP: desvio padrão; DCNT: doenças crônicas não transmissíveis.

^a^ QVRS- sem problemas = 1,000; qualquer problema < 1,000.

^b^ Informação faltante para 3 indivíduos.

^c^ Ter pelo menos um fator considerando atividade física insuficiente, consumo excessivo de bebidas alcoólicas, tabagismo ou consumo regular de alimentos não saudáveis.

Quando avaliados os domínios do EQ-5D-3L, verificou-se maior a frequência de algum problema de ansiedade/depressão aos 6 meses (44,9% *versus* 18,2%), problemas para realização de cuidados pessoais aos 12 meses (22,9% *versus* 8,0%) e atividades pessoais aos 12 meses (56,9% *versus* 22,0%) entre pacientes com síndrome pós-covid quando comparados aos sem essa condição (Tabela 4).

**Tabela 4:** Domínios de QVRSa segundo síndrome pós-covid-19 6 e 12 meses de alta hospitalar por covid-19. Cuiabá (MT), 2024.

Domínios	6 meses (n = 189)				p-valor	12 meses (n = 159)				p-valor
	Geral		Síndrome pós-covid-19^b^			Geral		Síndrome pós-covid-19^b^		
	n	%	Não n (%)	Sim n (%)		n	%	Não n (%)	Sim n (%)	
Mobilidade					< 0,01					0,23
Sem problema	155	82,1	22 (100,0)	133 (79,6)		121	76,1	41 (82,0)	80 (73,4)	
Algum problema	34	17,9	0 (0,0)	34 (20,4)		38	23,9	9 (18,0)	29 (26,6)	
Cuidados pessoais					0,78					0,02
Sem problema	178	94,2	21 (95,4)	157 (94,0)		130	81,8	46 (92,0)	84 (77,1)	
Algum problema	11	5,8	1 (4,5)	10 (6,0)		29	18,2	4 (8,0)	25 (22,9)	
Atividades habituais					0,14					< 0,01
Sem problema	161	85,2	21 (95,4)	140 (83,8)		86	54,1	39 (78,0)	47 (43,1)	
Algum problema	28	14,8	1 (4,5)	27 (16,2)		73	45,9	11 (22,0)	62 (56,9)	
Dor/mal-estar					0,06					0,72
Sem problema	131	69,3	19 (86,4)	112 (67,1)		86	54,1	26 (52,0)	60 (55,1)	
Algum problema	58	30,7	3 (13,6)	55 (32,9)		73	45,9	24 (48,0)	49 (44,9)	
Ansiedade/depressão					0,01					0,66
Sem problema	110	58,2	18 (81,8)	92 (55,1)		101	63,5	33 (66,0)	68 (62,4)	
Algum problema	79	41,8	4(18,2)	75 (44,9)		58	36,5	17 (34,0)	41 (37,6)	

QVRS: qualidade de vida relacionado à saúde.

^a^ QVRS- sem problemas = 1,000; qualquer problema < 1,000.

^b^ Síndrome pós-covid-19 classificada como a presença de algum sintoma persistentes após 12 semanas de alta hospitalar.

No modelo ajustado, síndrome pós-covid-19 manteve-se associada a ter qualquer problema na QVRS (RR = 2,43; IC95% 1,06–5,57) e ao domínio ansiedade/depressão (RR = 2,74; IC95% 1,08–7,01) aos 6 meses após a alta, essas associações não foram significativas quando avaliado os desfechos aos 12 meses da alta hospitalar (Tabela 5).

**Tabela 5:** Regressão de Poisson da associação do escore qualidade de vida relacionado a saúde (EQ-5D-3L) aos 6 e 12 meses da alta com síndrome pós-covid-19. Cuiabá (MT), 2024.

Domínios	Síndrome pós-covid (ref: não)	6 meses				12 meses			
		Não ajustada		Ajustada^b^		Não ajustada		Ajustada^c^	
		RR	(IC95%)	RR	(IC95%)	RR	(IC95%)	RR	(IC95%)
QVRS^a^									
	Sim	2,60	(1,19–5,70)	2,43	(1,06–5,57)	1,40	(0,93–2,10)	1,34	(0,88–2,04)
Mobilidade									
	Sim	-	-	-	-	5,32	(0,77–36,9)	3,48	(0,46–26,40)
Cuidados pessoais									
	Sim	1,32	(0,18–9,85)	0,29	(0,01–6,94)	1,94	(0,50–7,58)	1,41	(0,21–9,58)
Atividades habituais									
	Sim	3,55	(0,50–25,0)	0,78	(0,07–9,10)	1,95	(0,90–4,28)	1,53	(0,66–3,54)
Dor/mal-estar									
	Sim	2,41	(0,82–7,09)	1,32	(0,38–4,57)	1,95	(0,90–4,27)	1,81	(0,81–4,03)
Ansiedade/depressão									
	Sim	2,47	(1,00–6,10)	2,74	(1,08–7,0)	0,78	(0,45–1,34)	1,15	(0,64–2,09)

QVRS: qualidade de vida relacionado à saúde; IC95%: intervalo de confiança de 95%; DCNT: doenças crônicas não transmissíveis; RR: razão de risco.

^a^ QVRS- sem problemas = 1,000; qualquer problema < 1,000.

^b^ Ajustado por: sexo, faixa etária, escolaridade, raça/cor, número de comorbidades.

^c^ Ajustado por: sexo, faixa etária, escolaridade, raça/cor, número de comorbidades e coocorrência de fatores de risco para DCNT.

## DISCUSSÃO

Além da elevada proporção de pacientes internados por covid-19 que apresentaram síndrome pós-covid-19 e prejuízos na QVRS após 6 e 12 meses da alta hospitalar, os resultados deste estudo revelaram a associação independente da síndrome pós-covid-19 com pior QVRS após 6 meses da alta hospitalar. Ao avaliar os domínios da escala EQ-5D, verificou-se que a presença de síndrome pós-covid-19 elevou em mais de 2,7 vezes o risco de apresentar ansiedade/depressão aos 6 meses da alta hospitalar.

Os resultados deste estudo destacam a persistência dos impactos da covid-19 na qualidade de vida relacionada à saúde dos pacientes após a recuperação inicial. A alta proporção de pacientes que relataram pelo menos um sintoma da covid-19 após 6 e 12 meses da alta hospitalar destaca a natureza prolongada e debilitante da síndrome pós-covid-19 que continuam a afetar a vida dos pacientes muito além da fase aguda da infecção^7,9,17^.

Além disso, tem sido verificado que a duração da internação hospitalar e a gravidade da covid-19 na fase aguda, como a necessidade de ventilação mecânica, são fatores que contribuíram para a redução da QVRS após a recuperação^19^. Contudo, neste estudo, a síndrome pós-covid-19 permaneceu significativamente associada a pior QVRS mesmo após ajuste para fatores ligados a pior gravidade da covid-19, ressaltando o impacto duradouro dessa condição.

Foi elevada a proporção de indivíduos que relataram algum prejuízo na qualidade de vida aos 6 e 12 meses. Destaca-se o fato dessa proporção ter aumentado de 59,2% para 77,0% dos pacientes avaliados, respectivamente. Esses dados são alarmantes e evidenciam a necessidade urgente de estratégias de saúde pública como centros de reabilitação física e cognitiva, monitoramento e desenvolvimento de protocolos para acompanhamento dos sintomas persistentes e integração e capacitação da atenção primária para identificar casos de síndrome pós-covid-19.

A síndrome pós-covid-19 e a QVRS não se mantiveram associadas aos 12 meses após a alta neste estudo. No entanto, estudos realizados nos EUA^19^ após 24 meses, no Brasil^8^ após 12 meses e na China^15^ após 24 meses da alta hospitalar verificaram que sintomas persistentes estavam associados à diminuição da qualidade de vida durante o acompanhamento.

Nos EUA^19^, após 24 meses, sintomas persistentes foram associados a uma diminuição da qualidade de vida, com variabilidade significativa dependendo de fatores como idade, comorbidades e gravidade da infecção^19^. Na China^15^, sintomas como ansiedade e depressão impactaram a qualidade de vida, evidenciando que os efeitos da covid-19 vão além do período agudo^15^.

O estudo brasileiro de Ida et al.^8^ utilizou o SF-36 para avaliar a qualidade de vida, enquanto este estudo aplicou o EQ-5D-3L por domínios, o que pode especificar diferenças nos achados. As autoras verificaram que 67% dos pacientes internados apresentavam problemas na funcionalidade motora, e um terço deles não retornou ao trabalho após 12 meses. Além disso, foram identificadas alterações emocionais significativas, corroborando nossos resultados^8^.

Verifica-se, portanto, que a literatura que considerou síndrome pós-covid-19 em diferentes pontos do tempo verificaram tanto associações positivas com a qualidade de vida^19,22,27^ quanto a ausência de associação^28^.

Em estudo longitudinal com 24 meses de acompanhamento^19^, foi observado que os sintomas da síndrome pós-covid-19 atingem seu pico e a qualidade de vida atinge seu ponto mais baixo entre 6 e 12 meses após a infecção, o que se alinha com os achados deste estudo. Além disso, a proporção de pacientes relatando problemas de QVRS aumentou entre as avaliações de 6 e 12 meses, confirmando a tendência observada por Demko et al.^19^ de que o impacto na qualidade de vida é mais pronunciado durante esse período, devido à inflamação residual e fatores biológicos da doença e que podem piorar e exacerbar os sintomas persistentes. A literatura^11,17,19^ tem mostrado que a presença da síndrome pós-covid-19 está associada à diminuição da QVRS em domínios como funcionamento físico e saúde em geral. Este estudo mostrou que sintomas persistentes aos 6 meses foram associados ao domínio ansiedade/depressão. Esses achados são consistentes com a literatura que vem indicando que sintomas persistentes têm sido associados a níveis mais altos de sofrimento psicológico e estresse^17,19,27^.

Acredita-se que os efeitos da covid-19 no sistema nervoso estejam ligados ao receptor de enzima conversora da angiotensina 2–ECA^2^, presente em neurônios e no plexo coroide, embora em baixa quantidade. A neurofisiologia desses sintomas cognitivos não é bem compreendida, no entanto, fatores biológicos, como os efeitos da inflamação crônica gerada pela infeção, e fatores psicológicos, incluindo o medo e o impacto do isolamento social, podem interferir em funções essenciais do cérebro e diminuído a qualidade de vida^6,29^. Além disso, o apoio social é um preditor independente para menores sentimentos de ansiedade, menor risco de depressão e melhor percepção da qualidade de vida em pacientes com covid-19^29^.

O estudo prospectivo^19^destaca que diagnósticos de ansiedade e depressão pré-covid-19 não estavam associados à síndrome pós-covid-19, entretanto aqueles com esses diagnósticos no início do estudo apresentaram uma qualidade de vida consistentemente pior do que aqueles sem esses diagnósticos. Os achados da literatura^16,22,29,30^, que revelam uma alta prevalência de comprometimentos psiquiátricos e cognitivos, incluindo transtornos mentais comuns, depressão e ansiedade de 6 a 9 meses após a infecção por SARS-CoV-2, o que corrobora nossos resultados, que também identificaram impactos na saúde mental 6 meses após a infecção por covid-19, podendo estar relacionados à síndrome pós-covid-19 e resultar em prejuízos na qualidade de vida.

Este estudo acompanhou os mesmos indivíduos em dois momentos, aos 6 e 12 meses, com a adoção de questionário validado para avaliação da QVRS^21^. A taxa de seguimento aos 6 meses (73,3%) e 12 meses (62,5%) pode ser considerada elevada ao comparar com outros estudos brasileiros^8,18^ que tiveram porcentagens baixas de acompanhamento (entre 38,0% e 56,0%). Além disso, este estudo buscou analisar a associação independente da síndrome pós-covid-19 com a QVRS a partir de modelo de ajuste com base no diagrama causal, o que contribui para a compreensão dessa relação, uma vez que não foi verificado na literatura científica estudos que considerassem ajustes para potenciais fatores de confusão.

Entre as limitações deste estudo, deve ser destacada a amostragem por conveniência, que impede a generalização dos achados. A ausência de informações como os fatores de risco para DCNT antes da infecção por covid-19 e aos 6 meses também limitou a avaliação desses fatores como exposição pregressa a avaliação da qualidade de vida. Outra limitação desse estudo é o fato de não termos a avaliação da QVRS dos indivíduos antes da internação, para assim avaliar sua alteração após a internação por covid-19 e associação com a síndrome pós-covid-19. Além disso, não se pode ignorar o possível viés de memória para algumas perguntas retrospectivas e sobre os sintomas presentes na fase aguda durante a admissão hospitalar.

Em conclusão, nossos resultados sugerem que pacientes com síndrome pós-covid-19 apresentam prejuízos na QVRS até 6 meses após a alta hospitalar e maior chance de apresentar ansiedade/depressão aos seis meses da alta hospitalar. Esses resultados revelam os impactos a longo prazo nas funções físicas, emocionais e saúde mental das pessoas acometidas. Destaca-se a necessidade da ação ampla da atenção primária sobre os efeitos a longo prazo e seu impacto na QVRS, com estratégias de suporte psicológico e intervenções personalizadas podem para melhorar a qualidade de vida desses indivíduos e prevenir um agravamento dos sintomas mentais e físicos ao longo do tempo.

## Data Availability

Os conjuntos de dados gerados e/ou analisados durante o presente estudo não estão disponíveis publicamente devido a restrições [éticas/legais/de privacidade].
